# Supplementary material for: A new method for customized fetal growth reference percentiles
Source: PLoS One. 2023 Mar 16;18(3):e0282791. doi: 10.1371/journal.pone.0282791 (PMC10019672; doi:10.1371/journal.pone.0282791)
Supplement: S1 Fig — From the NICHD Fetal Growth Studies–Singletons. (DOCX) [file pone.0282791.s001.docx]

**Preterm:**

Delivered < 37 weeks (n=145)

**Missingness:**

Missing ultrasound data (n=5),

Missing model covariates (n=39)

**Abnormal pregnancy outcomes:**

Fetal anomalies (n=87),

Neonatal aneuploidy (n=5),

Missing pregnancy outcome (n=13)

**Ineligible and exclude:**

Ineligible after enrollment (n=18),

Deactivated (e.g., for pregnancy loss, moved, pregnancy termination, or lost to follow-up) (n=202)

Women staying

(n=2,332)

Women staying

(n=2,477)

Women staying

(n=2,582)

Final women

(n=2,288)

Women enrolled

(n=2,802)
