# Supplementary material for: A new method for customized fetal growth reference percentiles
Source: PLoS One. 2023 Mar 16;18(3):e0282791. doi: 10.1371/journal.pone.0282791 (PMC10019672; doi:10.1371/journal.pone.0282791)
Supplement: S1 Table — (DOCX) [file pone.0282791.s003.docx]

Supplement Table 1. Equations to calculate the percentiles for the Gardosi, heteroscedastic and quantile regression models.

| **Stages** | **Analysis** | **Gardosi Model** | **Heteroscedastic Model** | **Quantile Regression Model** |
| --- | --- | --- | --- | --- |
| **Stage 1** | **Evaluation of models** |  |  |  |
|  | Data set | Fetal Growth | Fetal Growth | Fetal Growth |
|  | Dependent | Birthweight | Birthweight | Birthweight |
|  | Independents | polynomials of six predictors | polynomials of six predictors | polynomials of six predictors |
|  | Models | regression model | regression model | regression model |
|  | Sigma and percentiles | Customized population mean, constant population CV and normal distribution assumption used to create the percentiles | Customized population mean and variance and normal distribution assumption used to create the percentiles | Using quantile regression model to create the percentiles |
|  | Comparisons | R2 determination coefficient, cv, plot of percentiles | R2 determination coefficient, cv, plot of percentiles | R2 determination coefficient, cv, plot of percentiles |
| **Stage 2** | **External validation of models** | |  |  |
|  | Data set | CSL | CSL | CSL |
|  | Outcome | Predicted birthweight | Predicted birthweight | Predicted birthweight |
|  | Predictors | polynomials of six predictors | polynomials of six predictors | polynomials of six predictors |
|  | Models | using regression coefficients from Fetal Growth | using regression coefficients from Fetal Growth | using regression coefficients from Fetal Growth |
|  | Sigma and percentiles | Customized population mean, constant population CV and normal distribution assumption used to create the percentiles | 'Customized population mean and variance and normal distribution assumption used to create the percentiles | Using quantile regression model to create the percentiles |
|  | Comparisons | Differences of actual and predicted values | Differences of actual and predicted values | Differences of actual and predicted values |
| **Stage 3** | **Misclassification of different LGA and SGA** | |  |  |
|  | Data set | CSL | CSL | CSL |
|  | Outcome | Neonatal Morbidity | Neonatal Morbidity | Neonatal Morbidity |
|  | Classification | different individual LGA and SGA | different individual LGA and SGA | different individual LGA and SGA |
|  | Comparisons | PPV, NPV, sensitivity, specificity, odds ratio and c-statistic | PPV, NPV, sensitivity, specificity, odds ratio and c-statistic | PPV, NPV, sensitivity, specificity, odds ratio and c-statistic |
