# Supplementary material for: A new method for customized fetal growth reference percentiles
Source: PLoS One. 2023 Mar 16;18(3):e0282791. doi: 10.1371/journal.pone.0282791 (PMC10019672; doi:10.1371/journal.pone.0282791)
Supplement: S3 Table — (DOCX) [file pone.0282791.s005.docx]

Supplemental Table 2. Customization coefficients for customized three models with the addition of paternal height and weight.

|  |  | **Gardosi Method** ^a^ | | | **Heteroscedastic Model** | | | **Quantile Regression Model (0.5)** | | |
| --- | --- | --- | --- | --- | --- | --- | --- | --- | --- | --- |
| **Variable** | | Estimate | SE | P | Estimate | SE | P | Estimate | SE | P |
|  | ***Mean Model*** |  |  |  |  |  |  |  |  |  |
|  | Intercept | 3479.946 | 40.080 | <.001 | 3480.000 | 40.001 | <.001 | 3450.685 | 41.294 | <.001 |
| Gestational age (from 280 d) | Linear term | -0.084 | 5.040 | 0.987 | -0.086 | 5.175 | 0.987 | -0.100 | 5.087 | 0.984 |
|  | Quadratic term | -0.908 | 0.454 | 0.046 | -0.914 | 0.461 | 0.048 | -1.250 | 0.449 | 0.006 |
|  | Cubic term | 0.001 | 0.061 | 0.993 | 0.004 | 0.068 | 0.958 | 0.005 | 0.065 | 0.941 |
| Sex | Male | 45.201 | 15.030 | 0.003 | 45.199 | 15.632 | 0.004 | 62.149 | 15.556 | <.0001 |
| Maternal height (from 163 cm) | Linear term | -1.513 | 3.959 | 0.703 | -1.517 | 4.045 | 0.708 | -3.992 | 4.323 | 0.356 |
|  | Quadratic term | 0.174 | 0.258 | 0.501 | 0.166 | 0.279 | 0.553 | 0.541 | 0.273 | 0.048 |
|  | Cubic term | 0.016 | 0.022 | 0.479 | 0.019 | 0.025 | 0.446 | 0.023 | 0.022 | 0.297 |
| Maternal prepregnancy weight (from 64 kg) | Linear term | 6.717 | 2.258 | 0.003 | 6.712 | 2.260 | 0.003 | 7.274 | 2.688 | 0.007 |
|  | Quadratic term | -0.345 | 0.128 | 0.007 | -0.385 | 0.129 | 0.003 | -0.336 | 0.157 | 0.033 |
|  | Cubic term | 0.003 | 0.002 | 0.149 | 0.004 | 0.002 | 0.036 | 0.003 | 0.002 | 0.204 |
| Race | Non-Hispanic black | -224.738 | 40.194 | <.001 | -224.738 | 37.882 | <.001 | -195.819 | 44.927 | <.001 |
|  | Hispanic | -110.699 | 41.207 | 0.007 | -110.699 | 44.235 | 0.012 | -83.917 | 42.536 | 0.049 |
|  | Asian/Pacific Islander | -97.758 | 51.477 | 0.058 | -97.758 | 46.755 | 0.037 | -32.676 | 58.164 | 0.574 |
| Parity | 1 | 60.223 | 33.525 | 0.073 | 60.222 | 32.979 | 0.068 | 37.155 | 38.365 | 0.333 |
|  | 2+ | 80.966 | 44.006 | 0.066 | 80.965 | 45.416 | 0.075 | 83.217 | 51.443 | 0.106 |
| Paternal height (from 177.8cm) | Linear term | 3.651 | 3.061 | 0.233 | 3.652 | 3.033 | 0.229 | 4.716 | 3.562 | 0.186 |
|  | Quadratic term | -0.334 | 0.174 | 0.055 | -0.347 | 0.161 | 0.030 | -0.155 | 0.190 | 0.415 |
|  | Cubic term | 0.003 | 0.011 | 0.774 | 0.002 | 0.011 | 0.878 | 0.007 | 0.017 | 0.686 |
| Paternal weight (from 81.6 kg) | Linear term | 3.029 | 1.550 | 0.051 | 3.029 | 1.553 | 0.051 | 4.157 | 1.912 | 0.030 |
|  | Quadratic term | -0.025 | 0.079 | 0.752 | 0.009 | 0.073 | 0.905 | -0.157 | 0.109 | 0.149 |
|  | Cubic term | 0.000 | 0.001 | 0.810 | -0.001 | 0.001 | 0.665 | 0.001 | 0.002 | 0.671 |
|  | ***Variance Model*** ^b^ |  |  |  |  |  |  |  |  |  |
|  | Intercept |  |  |  | 412.808 | 30.511 | <.001 |  |  |  |
| Gestational age (from 280 d) | Linear term |  |  |  | -0.025 | 0.021 | 0.223 |  |  |  |
|  | Quadratic term |  |  |  | -0.001 | 0.002 | 0.555 |  |  |  |
|  | Cubic term |  |  |  | 0.000 | 0.000 | 0.240 |  |  |  |
| Sex | Male |  |  |  | 0.114 | 0.060 | 0.056 |  |  |  |
| Maternal height (from 163 cm) | Linear term |  |  |  | -0.019 | 0.015 | 0.211 |  |  |  |
|  | Quadratic term |  |  |  | 0.000 | 0.001 | 0.688 |  |  |  |
|  | Cubic term |  |  |  | 0.000 | 0.000 | 0.835 |  |  |  |
| Maternal prepregnancy weight (from 64 kg) | Linear term |  |  |  | 0.007 | 0.007 | 0.333 |  |  |  |
|  | Quadratic term |  |  |  | 0.000 | 0.000 | 0.389 |  |  |  |
|  | Cubic term |  |  |  | 0.000 | 0.000 | 0.139 |  |  |  |
| Race | Non-Hispanic black |  |  |  | -0.216 | 0.152 | 0.157 |  |  |  |
|  | Hispanic |  |  |  | 0.075 | 0.140 | 0.591 |  |  |  |
|  | Asian/Pacific Islander |  |  |  | -0.368 | 0.201 | 0.067 |  |  |  |
| Parity | 1 |  |  |  | 0.119 | 0.125 | 0.339 |  |  |  |
|  | 2+ |  |  |  | 0.215 | 0.164 | 0.189 |  |  |  |
| Paternal height (from 177.8cm) | Linear term |  |  |  | -0.013 | 0.012 | 0.290 |  |  |  |
|  | Quadratic term |  |  |  | -0.001 | 0.001 | 0.061 |  |  |  |
|  | Cubic term |  |  |  | 0.000 | 0.000 | 0.301 |  |  |  |
| Paternal weight (from 81.6 kg) | Linear term |  |  |  | -0.004 | 0.006 | 0.558 |  |  |  |
|  | Quadratic term |  |  |  | 0.000 | 0.000 | 0.820 |  |  |  |
|  | Cubic term |  |  |  | 0.000 | 0.000 | 0.779 |  |  |  |

Note: 0.000 is used for any value <0.001.

**^a^** All three models included the same customizing variables containing cubic and quadratic terms of deviation of gestational time at delivery from the optimal 280 days mark a priori per the Gardosi model.^1^ In addition to the six proposed “physiological” variables (as designated by the Gardosi method) that influence fetal growth, models also included smoking, BMI (kg/m2), gestational diabetes, gestational hypertensive disease/preeclampsia, and antepartum bleeding. Analysis was centered on 280 days’ gestation, height 163 cm, prepregnancy weight 64 kg, nulliparous, and Non-Hispanic White race/ethnicity. However, only the coefficients for the six “physiologic” variables were included in an additive model to calculate the term optimal weight percentiles.

**^b^** Variance is only for the heteroscedastic model.

Supplemental Table 3. Comparison of model performance for the three different methods in detecting SGA and LGA with morbidity in the NICHD Fetal Growth Studies – Singletons (N=2,288).

| Classification | n | Composite neonatal morbidity  % | PPV^b^ | NPV | Sensitivity | Specificity | Odds ratio  (95% CI) | c-statistic  (95% CI) |
| --- | --- | --- | --- | --- | --- | --- | --- | --- |
| LGA > 90th |  |  |  |  |  |  |  |  |
| Duryea | 210 | 9.2 | 8.1 | 96.0 | 17.0 | 91.2 | 1.58 (0.88-2.82) | 0.52 (0.49-0.56) |
| Gardosi | 173 | 7.6 | 6.9 | 95.8 | 12.0 | 92.6 | 1.48 (0.77-2.81 | 0.52 (0.49-0.55) |
| Heteroscedastic | 223 | 9.8 | 7.6 | 96.0 | 17.0 | 90.6 | 1.60 (0.91-2.82) | 0.52 (0.49-0.56) |
| Quantile | 233 | 10.2 | 8.2 | 96.1 | 19.0 | 90.2 | 1.78 (1.04-3.05) | 0.53 (0.50-0.57) |
|  |  |  |  |  |  |  |  |  |
| SGA < 10th |  |  |  |  |  |  |  |  |
| Duryea | 197 | 8.6 | 4.6 | 96.4 | 10.6 | 91.5 | 0.90 (0.41-1.98) | 0.50 (0.48-0.53) |
| Gardosi | 130 | 5.7 | 4.6 | 96.3 | 7.1 | 94.4 | 0.99 (0.39-2.48) | 0.50 (0.48-0.52) |
| Heteroscedastic | 203 | 8.9 | 3.9 | 96.3 | 9.4 | 91.1 | 0.87 (0.40-1.91) | 0.51 (0.48-0.53) |
| Quantile | 219 | 9.6 | 4.6 | 96.4 | 11.8 | 90.5 | 0.80 (0.37-1.75) | 0.51 (0.48-0.54) |
|  |  |  |  |  |  |  |  |  |
| SGA < 5th |  |  |  |  |  |  |  |  |
| Duryea | 92 | 4.0 | 6.5 | 96.4 | 7.1 | 96.1 | 1.45 (0.57-3.65) | 0.51 (0.48-0.53) |
| Gardosi | 56 | 2.5 | 8.9 | 96.4 | 5.9 | 97.7 | 1.94 (0.69-5.50) | 0.51 (0.49-0.53) |
| Heteroscedastic | 97 | 4.2 | 6.2 | 96.4 | 7.1 | 95.9 | 1.36 (0.54-3.44) | 0.51 (0.48-0.53) |
| Quantile | 110 | 4.8 | 4.6 | 96.3 | 5.9 | 95.2 | 1.00 (0.40-2.51) | 0.50 (0.50-0.50) |

Note: In the Consortium on Safe Labor Study, birthweight was predicted by using models' coefficients from the NICHD Fetal Growth Studies. Large- and small-for-gestational-age (LGA and SGA, respectively) were defined according to different models, then calculated the positive predictive value (PPV), negative predictive value (NPV), sensitivity, specificity, odds ratio and c-statistic using neonatal morbidity as the outcome. Neonatal morbidities were selected specifically for SGA or LGA based on increased risks associated with these and included: metabolic acidosis (pH <7.1 and base deficit >12mmol/L), NICU stay greater than three days, pneumonia, respiratory distress syndrome, persistent pulmonary hypertension, seizures, hyperbilirubinemia requiring exchange transfusion, intrapartum aspiration (meconium, amniotic fluid, blood), neonatal death, mechanical ventilation at term, necrotizing enterocolitis, hypoglycemia, hypoxic ischemic encephalopathy, periventricular leukomalacia (SGA only), sepsis based on blood culture (SGA only), bronchopulmonary dysplasia/chronic lung disease (SGA only), retinopathy of prematurity (SGA only), and birth injury (LGA only).^2-6^

Supplement Table 4. Equations to calculate the percentiles for the Gardosi, heteroscedastic and quantile regression models.

| **Stages** | **Analysis** | **Gardosi Model** | **Heteroscedastic Model** | **Quantile Regression Model** |
| --- | --- | --- | --- | --- |
| **Stage 1** | **Evaluation of models** |  |  |  |
|  | Data set | Fetal Growth | Fetal Growth | Fetal Growth |
|  | Dependent | Birthweight | Birthweight | Birthweight |
|  | Independents | polynomials of six predictors | polynomials of six predictors | polynomials of six predictors |
|  | Models | regression model | regression model | regression model |
|  | Sigma and percentiles | Customized population mean, constant population CV and normal distribution assumption used to create the percentiles | Customized population mean and variance and normal distribution assumption used to create the percentiles | Using quantile regression model to create the percentiles |
|  | Comparisons | R2 determination coefficient, cv, plot of percentiles | R2 determination coefficient, cv, plot of percentiles | R2 determination coefficient, cv, plot of percentiles |
| **Stage 2** | **External validation of models** | |  |  |
|  | Data set | CSL | CSL | CSL |
|  | Outcome | Predicted birthweight | Predicted birthweight | Predicted birthweight |
|  | Predictors | polynomials of six predictors | polynomials of six predictors | polynomials of six predictors |
|  | Models | using regression coefficients from Fetal Growth | using regression coefficients from Fetal Growth | using regression coefficients from Fetal Growth |
|  | Sigma and percentiles | Customized population mean, constant population CV and normal distribution assumption used to create the percentiles | 'Customized population mean and variance and normal distribution assumption used to create the percentiles | Using quantile regression model to create the percentiles |
|  | Comparisons | Differences of actual and predicted values | Differences of actual and predicted values | Differences of actual and predicted values |
| **Stage 3** | **Misclassification of different LGA and SGA** | |  |  |
|  | Data set | CSL | CSL | CSL |
|  | Outcome | Neonatal Morbidity | Neonatal Morbidity | Neonatal Morbidity |
|  | Classification | different individual LGA and SGA | different individual LGA and SGA | different individual LGA and SGA |
|  | Comparisons | PPV, NPV, sensitivity, specificity, odds ratio and c-statistic | PPV, NPV, sensitivity, specificity, odds ratio and c-statistic | PPV, NPV, sensitivity, specificity, odds ratio and c-statistic |

References

1. Gardosi J, Francis A. A customized standard to assess fetal growth in a US population. Am J Obstet Gynecol 2009;201:25 e1-7.

2. Longo S, Bollani L, Decembrino L, Di Comite A, Angelini M, Stronati M. Short-term and long-term sequelae in intrauterine growth retardation (IUGR). The journal of maternal-fetal & neonatal medicine : the official journal of the European Association of Perinatal Medicine, the Federation of Asia and Oceania Perinatal Societies, the International Society of Perinatal Obstet 2013;26:222-5.

3. Rosenberg A. The IUGR newborn. Seminars in perinatology 2008;32:219-24.

4. Giapros V, Drougia A, Krallis N, Theocharis P, Andronikou S. Morbidity and mortality patterns in small-for-gestational age infants born preterm. The journal of maternal-fetal & neonatal medicine : the official journal of the European Association of Perinatal Medicine, the Federation of Asia and Oceania Perinatal Societies, the International Society of Perinatal Obstet 2012;25:153-7.

5. McIntire DD, Bloom SL, Casey BM, Leveno KJ. Birth weight in relation to morbidity and mortality among newborn infants. The New England journal of medicine 1999;340:1234-8.

6. King JR, Korst LM, Miller DA, Ouzounian JG. Increased composite maternal and neonatal morbidity associated with ultrasonographically suspected fetal macrosomia. The journal of maternal-fetal & neonatal medicine : the official journal of the European Association of Perinatal Medicine, the Federation of Asia and Oceania Perinatal Societies, the International Society of Perinatal Obstet 2012;25:1953-9.
